# Global prevalence of intimate partner violence during the COVID-19 pandemic among women: systematic review and meta-analysis

**DOI:** 10.1186/s12905-023-02845-8

**Published:** 2024-02-17

**Authors:** Mearg Eyasu Kifle, Setognal Birara Aychiluhm, Etsay Woldu Anbesu

**Affiliations:** 1 Family Guidance Association, Logia, Afar Ethiopia; 2 Department of Public Health, College of Medical and Health Sciences, Samara University, Samara, Ethiopia

**Keywords:** COVID-19, Intimate partner violence, Women, Pooled prevalence

## Abstract

**Background:**

During the coronavirus pandemic, people faced strict preventive measures, including staying at home and maintaining social distance, which led to increasing rates of intimate partner violence. Women have been facing dual health emergencies, including COVID-19 and domestic violence. Despite this, there is a lack of representative data on intimate partner violence during the COVID-19 pandemic and inconsistent findings.

**Methods:**

The Preferred Reporting Items for Systematic Reviews and Meta-Analyses guidelines were used to develop the systematic review and meta-analysis. All English-language studies conducted between 31 December 2019 and May 15/2022 were extracted from databases such as PubMed/Medline, CINAHL, and Google Scholar. The quality of the articles was assessed using the Joanna Briggs Institute Meta-Analysis of Statistics Assessment and Review Instrument (JBI-MAStARI). The I^2^ was used to assess heterogeneity among studies. Publication bias was assessed using funnel plot inspection and Egger’s test. A random effect model was used for the analysis using RevMan and STATA 14 software.

**Result:**

A total of 5065 studies were retrieved, and 14 studies were included in the final meta-analysis. The pooled prevalence of intimate partner violence was 31% (95% CI: 22, 40). Subgroup analysis based on region showed that the highest prevalence of intimate partner violence was in developing regions (33, 95% CI: 23.0, 43.0) compared to developed regions (14, 95% CI: 11.0, 17.0). Subgroup analysis based on country showed that Uganda had the highest prevalence of IPV 68% (95% CI: 62.0, 72.0), and the lowest was in the USA 10% (95% CI: 7.0, 15.0).

**Conclusion:**

Nearly one in three women experienced intimate partner violence during the COVID-19 pandemic. Subgroup analysis based on region showed that the highest prevalence of intimate partner violence was in developing regions (33%). All forms of intimate partner violence (physical, sexual, emotional, and economic) were prevalent. Thus, available interventions should be implemented to alleviate women’s intimate partner violence during the COVID-19 pandemic and similar emerging and remerging pandemics, particularly in developing countries.

**Trial registration:**

PROSPERO registration number: CRD42022334613.

**Supplementary Information:**

The online version contains supplementary material available at 10.1186/s12905-023-02845-8.

## Introduction

Gender-based violence (GBV) is any cruelty directed at individuals based on their sex, gender identity, or socially defined way of femaleness and maleness [1–3]. Violence against women is the primary form of GBV and is a basic violation of women’s human rights [1–5]. Threats, coercion, and denial of liberty against women are some of the violence against women [5–7]. The main actors of violence against women are male partners, including husbands, fiancées, or ex-partners, often referred to as intimate partners [5–8]. The World Health Organization (WHO) defines intimate partner violence as any behaviour within an intimate relationship by an intimate partner that causes physical, psychological, and sexual harm to those in the relationship, and it is one of the most common types of violence experienced by women [7–9].

Intimate partner violence is a serious, highly prevalent, preventable public health problem that violates women’s rights [10]. It has been exacerbated during the COVID-19 pandemic following control and prevention actions such as isolation, stay-at-home, and movement restrictions, targeted at reducing the pandemic have brought vulnerable women and potential perpetrators under the confines of the home setting and have increased the risk of IPV [11–13]. Globally, one in three women experiences physical, sexual, or psychological harm from an intimate partner or ex-partner [14, 15]. The World Health Organization (WHO) and European Commission evidence indicated a ‘shadow pandemic’, with the strong potential of increased IPV across the globe as seen during the Ebola pandemic. At the beginning of the pandemic (March–April), community-based victim organizations reported a 25–50% increase in hotline calls, up to a 150% increase in website traffic, and a 12.5% increase in IPV-related police activity [16–19].

Lockdown declarations following the COVID-19 pandemic in several countries of developed countries increased intimate partner violence by 20%, 21–35%, 32–36%, and 30–50% [12, 20]. In Africa, approximately 36.6% of women experience lifetime physical or sexual IPV [7]. During the COVID-19 pandemic, in Kenya (35%), Somalia (50%), South Africa (10660), Niger (499 cases) and Ethiopia (12.9%), Intimate partner violence has been reported to be as high as before [21]. In Ethiopia, more than 100 girls have been raped during COVID-19 within less than 2 months, and some of them are close family members [13].

Intimate partner violence has a complex and multifaceted health outcome, including physical, mental, sexual, and reproductive health issues, which, in turn, result in a high degree of women’s morbidity and mortality [22]. A study performed by the WHO showed that women who experienced violence were twice as likely to have an abortion and doubled their likelihood of falling into depression [23]. Approximately 41% of female IPV survivors experience some form of physical injury [24]. IPV can also extend beyond physical injury and result in death. Data from U.S. crime reports suggest that 16% of murder victims are killed by an intimate partner and that over 40% of female homicide victims in the U.S. are killed by an intimate partner [25].

There are policies and strategies implemented to overcome the problem at the global or local level just before and after the pandemic, including teaching safe and healthy relationship skills, engaging influential adults and peers, disrupting developmental pathways toward IPV, creating protective environments, strengthening economic support for families, and supporting survivors. Increased safety and lessened harm, commitment, cooperation, and leadership from numerous sectors, including public health, education, justice, health care, social services, business and labor, and government [26–31]. Despite this intervention, intimate partner violence remains a major public health problem during the COVID-19 pandemic. Moreover, there is a lack of representative data on intimate partner violence during the COVID-19 pandemic and inconsistent findings. Therefore, this systematic review and meta-analysis aimed to estimate the pooled prevalence of intimate partner violence during the COVID-19 pandemic among women.

## Methods

### Protocol and registration

These systematic reviews and meta-analyses were registered with the International Prospective Register of Systematic Reviews PROSPERO with an ID number (CRD42022334613) available at https://www.crd.york.ac.uk/prospero/#myprospero.

### Form of violence

**Physical violence** includes slapping, hitting, kicking and beating.

**Sexual violence** includes forced sexual intercourse and other forms of sexual coercion.

**Emotional (psychological) abuse** includes insults, belittling, constant humiliation, intimidation (e.g., destroying things), threats of harm, and threats to take away children.

**Controlling behaviours**, including isolating a person from family and friends; monitoring their movements; and restricting access to financial resources, employment, education or medical care.

### Search strategy and appraisal of studies

All published studies conducted in different countries that reported intimate partner violence during COVID-19 from December 2019 to May 2022 were included. The search was limited to peer-reviewed, indexed scientific journals and written in English. “The Preferred Reporting Items for Systematic Reviews and Meta-Analyses (PRISMA) guidelines [32] were used to develop the present systematic review using PRISMA checklist − 2020 (supplementary material file 1).

Article searches were conducted in databases including PubMed/MEDLINE, CINAHL, and Google Scholar. Medical Subject Headings (MeSH) terms and entry terms were used to search studies, and amendments were made based on the types of databases. The key terms and entry terms were connected by Boolean operators (supplementary material file 2). Screening was conducted independently by both authors (ME and EW), and disagreements were resolved through discussion with the third author (SB). Using a snowballing technique, references of eligible studies and relevant reviews were also searched.

### Eligibility criteria

The study included studies performed in different countries (globally), observational study designs included cross-sectional and cohort studies, published and unpublished studies, studies that reported the prevalence of intimate partner violence during COVID-19, only quantitative results for studies that reported both quantitative and qualitative results, English published articles, women having intimate partner violence, and studies conducted since COVID-19 identified in Wuhan, China from December 2019 to May 2022 (databases search date may 15–30/2022) were included. However, studies other than English, articles with no available full text and no response for relevant missing data after email contact with the corresponding author, case and conference reports, reviews, letters, and qualitative results for studies that reported both quantitative and qualitative results were excluded.

### CoCoPop/PEO

***Condition*** *:* Women’s intimate partner violence during the COVID-19 pandemic.

**Context:** worldwide.

***Population:*** women with partners.

***Exposure of interest*** *:* exposure is a determinant that increases or decreases the likelihood of intimate partner violence during the COVID-19 pandemic. The determinants can be but are not limited to age, residence, husbands’ educational status, decision-making power, social support, wealth index, history of abortion, arranged marriage, history of child death, controlling behaviour of the husband, and COVID-19 pandemic.

***Outcome/condition*** **:** The outcome of the study was the pooled prevalence of intimate partner violence during COVID-19. Intimate partner violence includes physical, sexual, and emotional abuse and controlling behaviours by an intimate partner [7].

### Study selection

Two independent reviewers (ME and EW) screened the searched studies. Duplicate articles were removed, assessments of articles using their titles and abstracts were performed, and irrelevant titles and abstracts were removed. A full-text review of relevant studies was performed before the inclusion of studies in the final meta-analysis. Disagreements among reviewers during the review process were resolved through discussion with the third author (SB). Endnote reference manager software [33] was used to collect and remove duplicate, irrelevant titles and abstracts.

### Quality assessment

During the screening process, two independent reviewers (ME and EW.) performed the quality assessment and evaluated the risk of bias in eligible studies. The “Joanna Briggs Institute Meta-Analysis of Statistics Assessment and Review Instrument (JBI-MAStARI)” tool was used to critically appraise the quality of the studies (supplementary material file 3) [34]. The components of the quality assessment were clear inclusion criteria, study population and setting**,** measurement criteria, event and exposure measurements, and appropriate statistical analysis*.* During the critical quality appraisal of the studies, any disagreement among the authors was resolved by discussion with the third author (SB).

### Data extraction

Data were extracted independently by two authors (ME and EW) using a pilot test data extraction Excel sheet and RevMan software. The outcome data extraction format contains the authors’ names, publication year, countries, study design, study setting, and sample size. Any disagreement was resolved through discussion with the third author (SB). In the case of incomplete results, email contact with the corresponding author was made, and articles were excluded if no response was made.

### Statistical analysis

The final included studies were imported to STATA version 14 to determine the pooled prevalence. The results were reported in narrative descriptions, tables, and graphs. A random-effects model was used to estimate the true effect at the 95% CI [35].

The results were reported using a forest plot with respective odds ratios and 95% CIs. Heterogeneity among the included studies was assessed by visual graphical inspection of the forest plot [36] and statistically using the I^2^ statistic [37]. I^2^ statistics of 25, 50, and 75% indicated low, moderate, and increased levels of heterogeneity, respectively, with *p* < 0.05.

Publication bias was identified using visual inspection of the funnel plot. In addition, evidence of publication bias was assessed statistically using Egger’s tests [38] at *p* < 0.05. The differences in heterogeneity between the studies were performed by subgroup analysis and meta-regression [39] based on country, study area (developed/developing), and sample size.

## Results

### Study selection

**Fig. 1:**
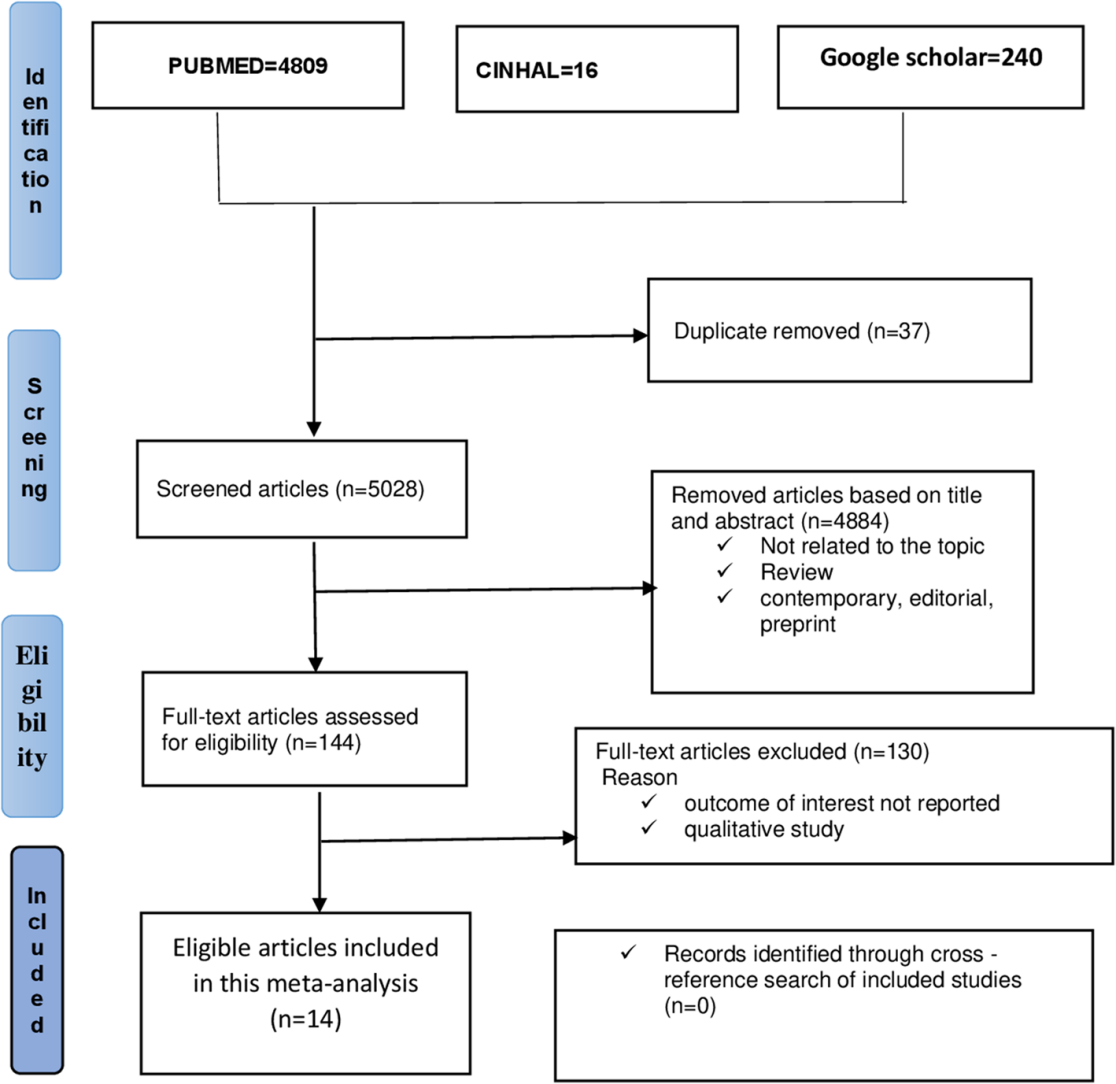
Flow chart of study selection for meta-analysis of IPV among women during the COVID-19 pandemic, 2022

A total of 5065 articles were collected from PubMed/MED-LINE, CINAHL, and Google Scholar. All articles were imported into EndNote software (version X8; Thomson Reuters, New York, NY), and 37 articles were excluded due to duplications. A total of 4884 articles were excluded after a review of their titles and abstracts. A total of 144 articles were assessed for eligibility based on the preset criteria. A total of 130 articles were excluded because the outcome of interest was not reported, and qualitative studies were excluded. Finally, 14 articles were eligible and included in this meta-analysis (Fig. 1).

### Quality appraisal

All the included studies met a minimum of four out of eight (50% and above) JBI critical appraisal scores. The criteria for inclusion were clearly defined in all studies. Strategies to address confounding factors and appropriate statistics were made in all included studies. However, since all the included studies were cross-sectional studies, the identification of confounding factors was not applicable for this study (supplementary material file 4).

### Characteristics of included studies

All 14 studies in this systematic review and meta-analysis were cross-sectional studies; eight of them were conducted in Ethiopia [4, 13, 40–45], one was conducted in Congo [46], one was conducted in Uganda [47], one was conducted in Bangladesh [48], one was conducted in Arab countries [49], one was conducted in Canada [50], and one was conducted in the USA [51]. A total of 8335 women with intimate partners were involved in our study. The sample size of the studies ranged from 216 [50] to 2002 [46]. In this review, the lowest prevalence (7.1%) of intimate partner violence was in St. Paul’s Hospital, Ethiopia [45], while the highest prevalence (68%) of IPV was reported in Uganda 48 (Table 1).

**Table 1:** Descriptive Characteristics of the included Studies (*n* = 14)

Author/s Year of Publication (ref)	Study area	Country	Study design	Sample size	Response rate (%)	Prevalence (%)	Study subjects
Cannon et al. (2021) [51]	Online survey	USA	Cross - sectional	279	100	74	Reproductive age
Ditekemena JD et al. (2021) [46]	Online survey	Congo	Cross - sectional	2002	100	11.7	Reproductive age
EHITEMARIYAM [4]	Debreberhan Town	Ethiopia	Cross - sectional	796	100	42.3	Reproductive age
El-Nimr NA et al. (2021) [49]		Arab countries	Cross - sectional	490	100	49.2	Married women
G Fetene et al. (2022) [41]	Bench Sheko zone	Ethiopia	Cross - sectional	590	99.3	39.2	Pregnant women
Gebrewahd GT et al. (2020) [40]	Aksum town	Ethiopia	Cross - sectional	682	100	24.6	Reproductive age
Katushabe E et al. (2022) [47]	Mbarara City Health Centre IV	Uganda	Cross - sectional	345	100	67.5	Pregnant women
Muldoon KA et al. (2021) [50]	Ottawa Hospital	Canada	Cross - sectional	216	42.6	24.07	Postpartum women
Wondale Getnet et al. (2022) [42]	Gondar	Ethiopia	Cross - sectional	804	95	48.6	Reproductive age
Rayhan I & Khaleda Akter (2021) [48]		Bangladesh	Cross - sectional	510	84.3	45.3	Married women
Shewangzaw Engda A et al. (2022) [43]	Debre- Berhan town	Ethiopia	Cross - sectional	700	95.1	19	Reproductive age
Shitu S et al. (2021) [13]	Gurage Zone	Ethiopia	Cross - sectional	448	96.9	24.1	Reproductive age
Tadesse AW et al. (2020) [44]	Dessie	Ethiopia	Cross - sectional	589	95.5	22.4	Married women
Teshome A et al. (2021) [45]	St. Paul’s Hospital	Ethiopia	Cross - sectional	464	100	7.1	Prenatal care attendant

### Pooled prevalence of any form of intimate partner violence among women during COVID-19

**Fig. 2:**
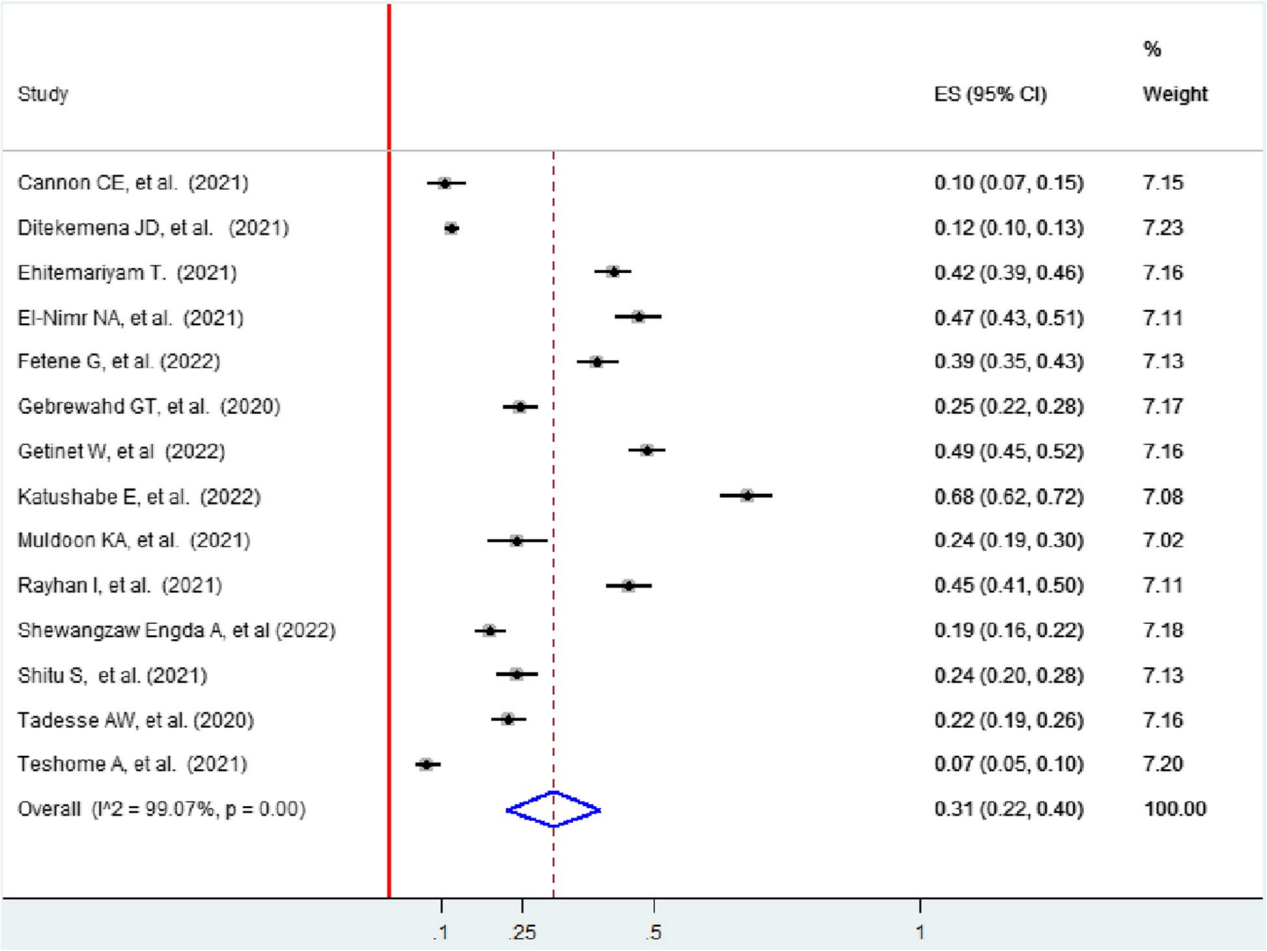
Forest plot showing the pooled prevalence of IPV among women during the COVID-19 pandemic

The pooled prevalence of intimate partner violence among women was 31% (95% CI: 22, 40)). In this review, the lowest prevalence (7.1%) of IPV was in St. Paul’s Hospital, Ethiopia [45], while the highest prevalence (68%) of intimate partner violence was reported in Uganda [51]. The included studies exhibited significant heterogeneity (I^2^ = 99.07, *p* < 0.001) (Fig. 2).

### Subgroup analysis

**Fig. 3:**
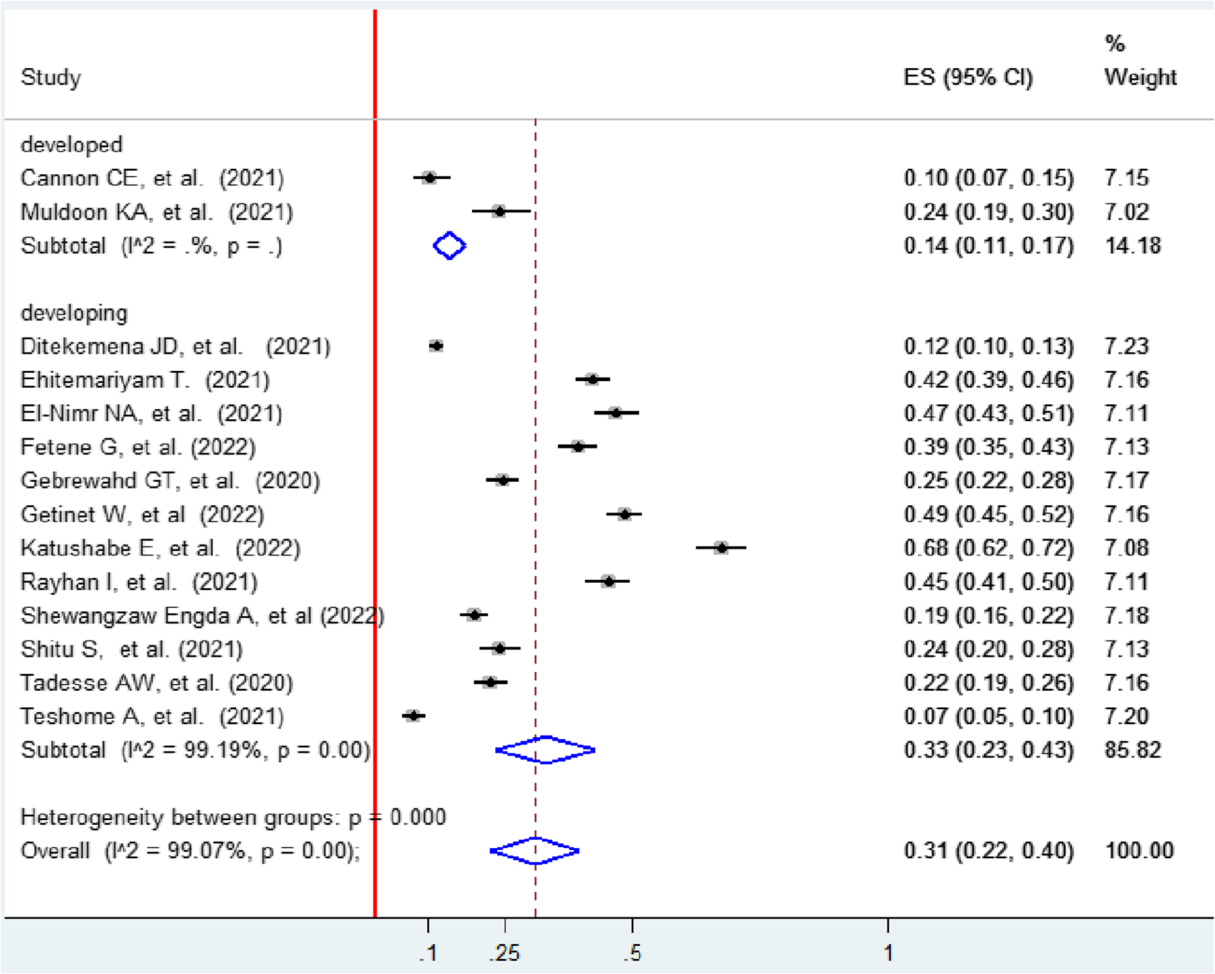
Forest plot showing subgroup analysis on IPV among women during the COVID-19 pandemic by region

Subgroup analysis was performed based on region (developed/developing) and country to identify the possible source of heterogeneity. Subgroup analysis based on region showed that the highest prevalence of intimate partner violence was in developing regions (33, 95% CI: 23.0, 43.0) compared to developed regions (14, 95% CI: 11.0, 17.0). High heterogeneity was reported in developing countries (I 2 = 99.19; *p* < 0.001) (Fig. 3).

**Fig. 4:**
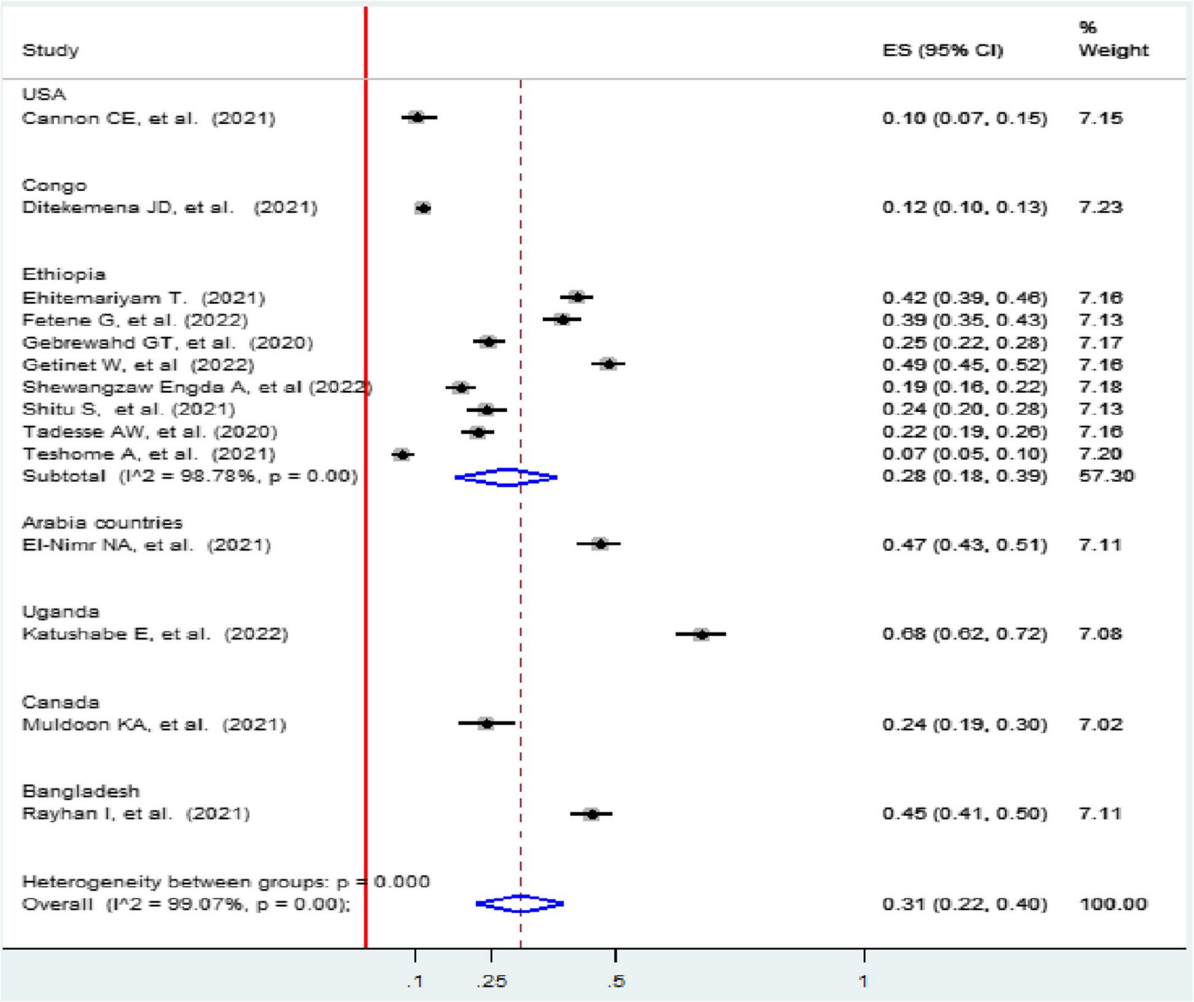
Forest plot subgroup prevalence of IPV among women during the COVID-19 pandemic by country

Subgroup analysis based on country showed that Uganda had the highest prevalence of intimate partner violence among women (68, 95% CI: 62.0, 72.0), and the lowest was in the USA (10, 95% CI: 7.0, 15.0%). High heterogeneity was reported in studies performed in Ethiopia (I 2 = 98.78; *p* < 0.001). Ethiopia had the highest weight of 57.30, and the possible reason may be the high number of studies performed and included in that area, and the lowest weight was in Canada, 7.02 (Fig. 4).

### Metaregression

Meta-regression was performed to identify the source of heterogeneity across the studies by considering continuous and categorical variables, including region (developed/developing), country, and sample size. Meta-regression indicated that no heterogeneity was observed (*p* value> 0.05) (Table 2).

**Table 2:** Meta-regression by country, region, and sample size

logprop	exp(b)	Std. Err.	t	p > |t|	[95% Conf. Interval]	
region_cat	2.361253	1.268853	1.60	0.141	.7131026	7.818673
country_cat	.9282646	.1058783	−0.65	0.529	.7199446	1.196863
Samplesize	.9993824	.0004416	−1.40	0.192	.998399	1.000367
_cons	.1096193	.117626	−2.06	0.066	.0100355	1.197389

**Fig. 5:**
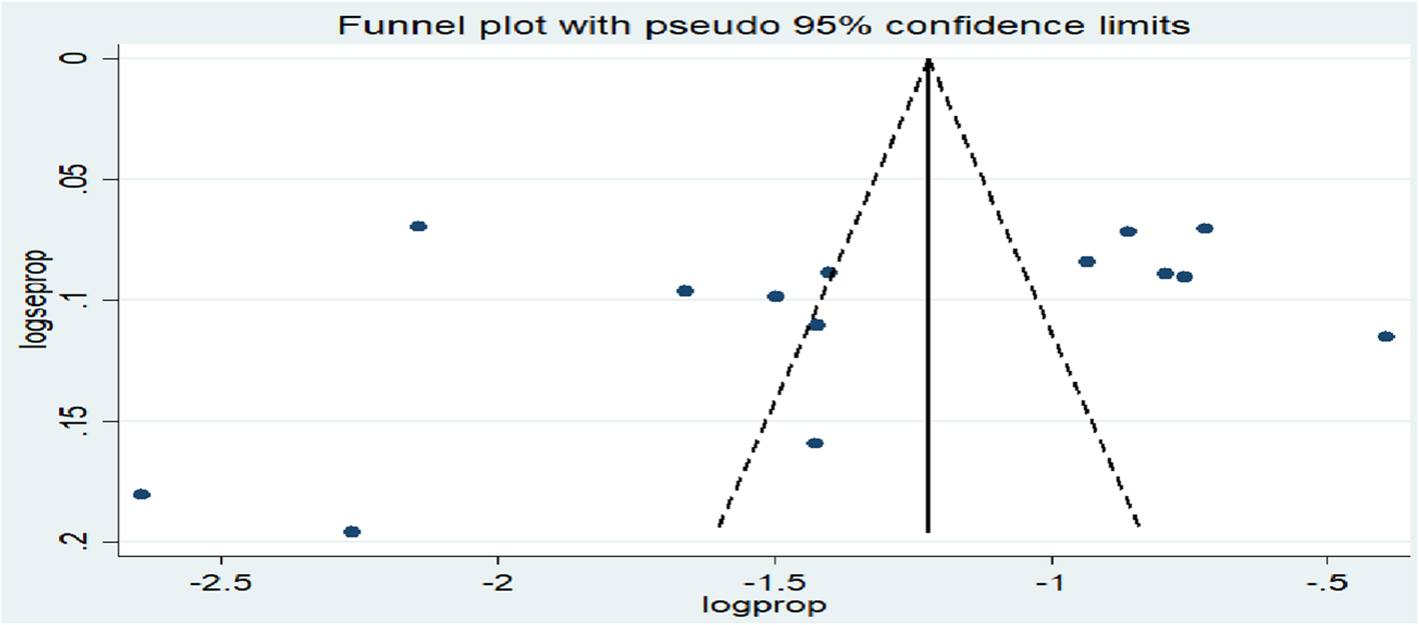
Funnel plot for publication bias, IPV among women during the COVID-19 pandemic

**Publication bias:** On visual inspection, asymmetry was observed in the funnel plots since there were six studies on the right and eight studies on the left (Fig. 5). However, the results from Egger’s regression test did not show statistical significance (*p* = 0.345) (Table 3).

**Table 3:** Egger’s test of publication bias and IPV among women during the COVID-19 pandemic

Std_Eff	Coef.	Std. Err.	t	p > |t|	[95% Conf. Interval]	
slope	−.6885023	.5631338	−1.22	0.245	−1.915465	.5384608
bias	−5.887438	5.986692	−0.98	0.345	−18.93132	7.156442

### Sensitivity analysis

The results showed that no single study unduly influenced the overall estimate of intimate partner violence during the COVID-19 pandemic and its associated factors (supplementary Figure file S1)*.*

### Forms of intimate partner violence

In this study, the prevalence was calculated for each form of intimate partner violence.

### Controlling violence

**Fig. 6:**
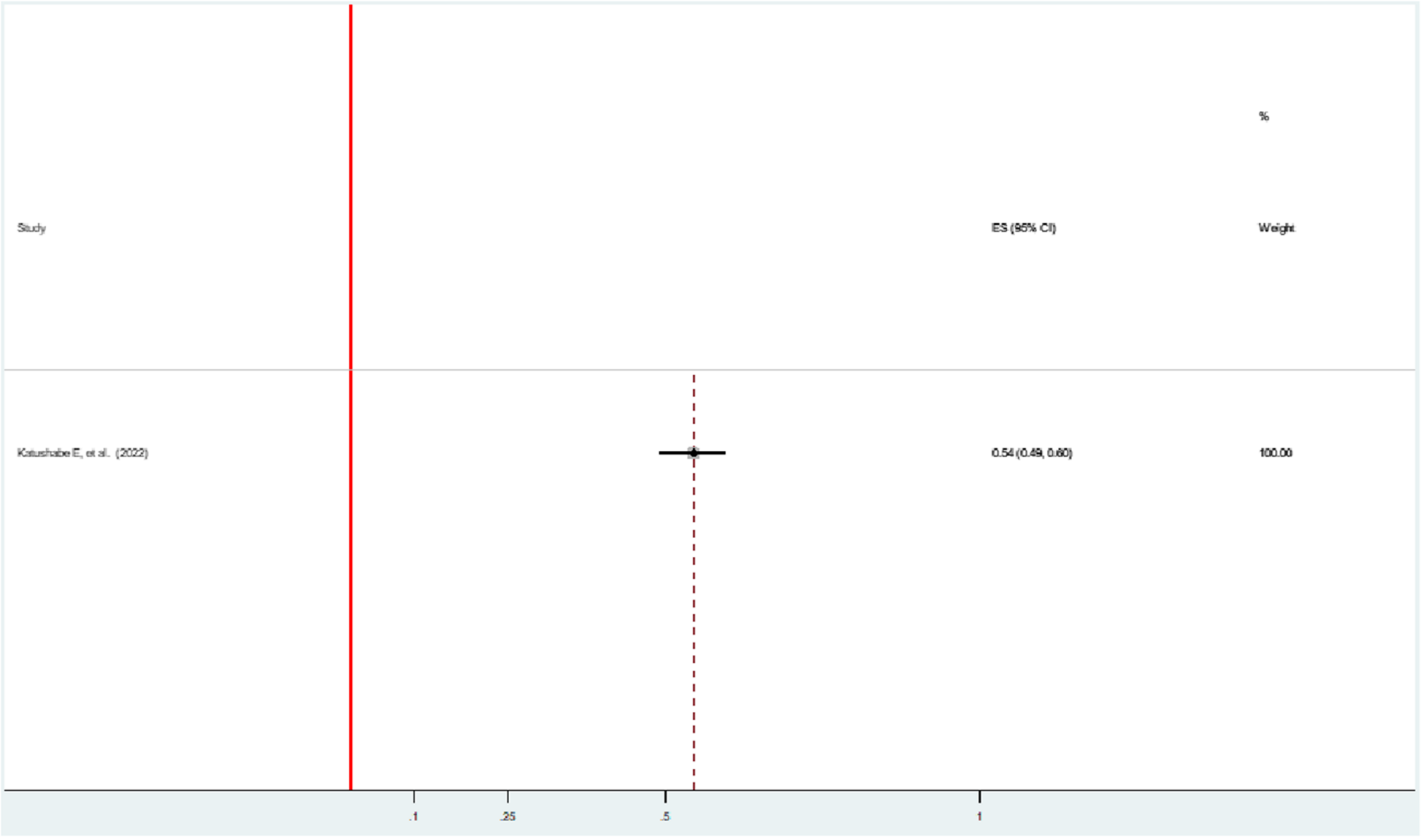
Forest plot showing the prevalence of controlling violence among women during the COVID-19 pandemic

The prevalence of controlling violence in one study during the pandemic was 54% (95% CI: 49, 60) [47] (Fig. 6).

### Verbal violence

**Fig. 7:**
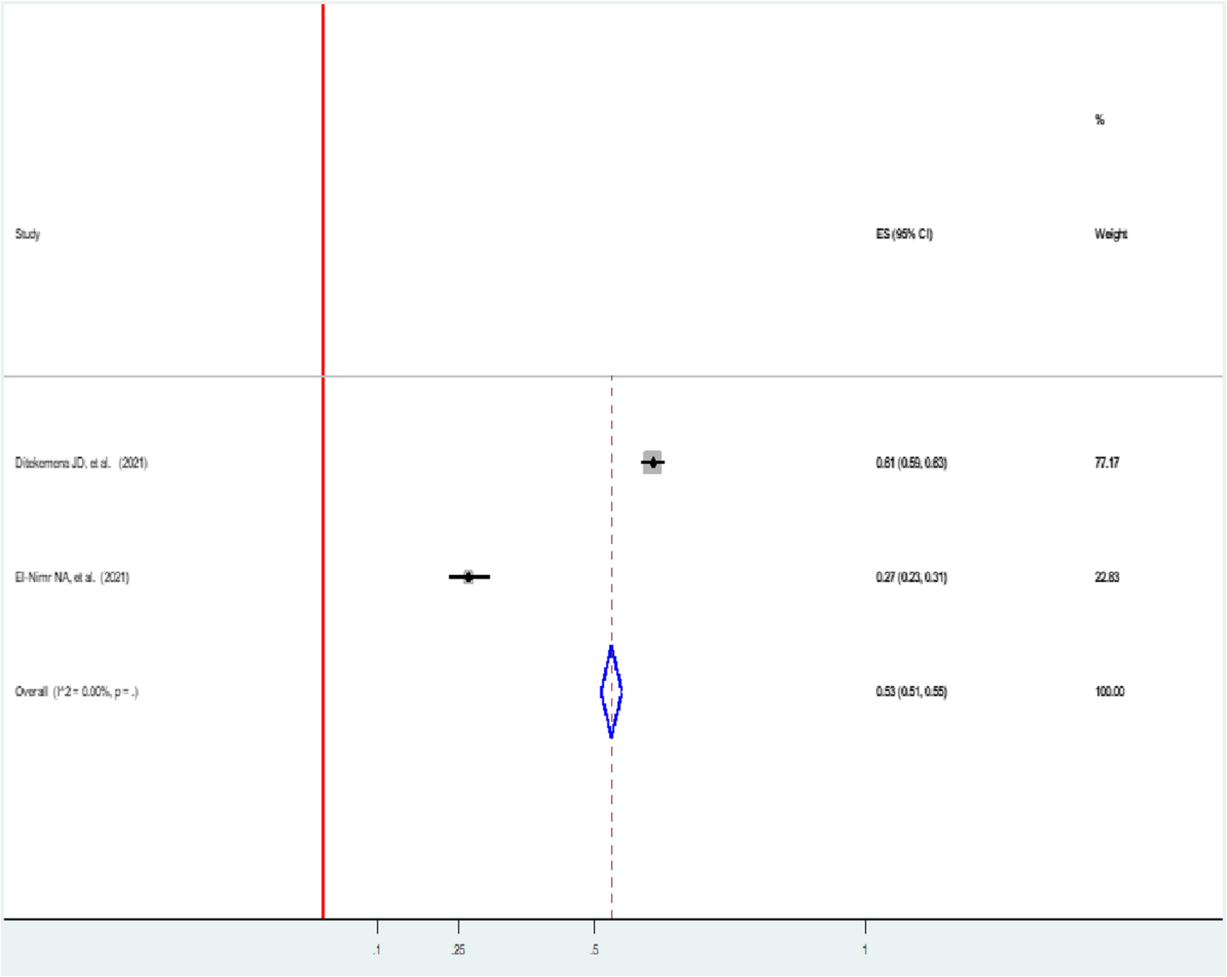
Forest plot showing the pooled prevalence of verbal among women during the COVID-19 pandemic

The pooled prevalence of verbal violence faced by women with intimate partners during the pandemic in two studies was 53% (95% CI: 51, 56) [46, 49] (Fig. 7).

### Emotional violence

**Fig. 8:**
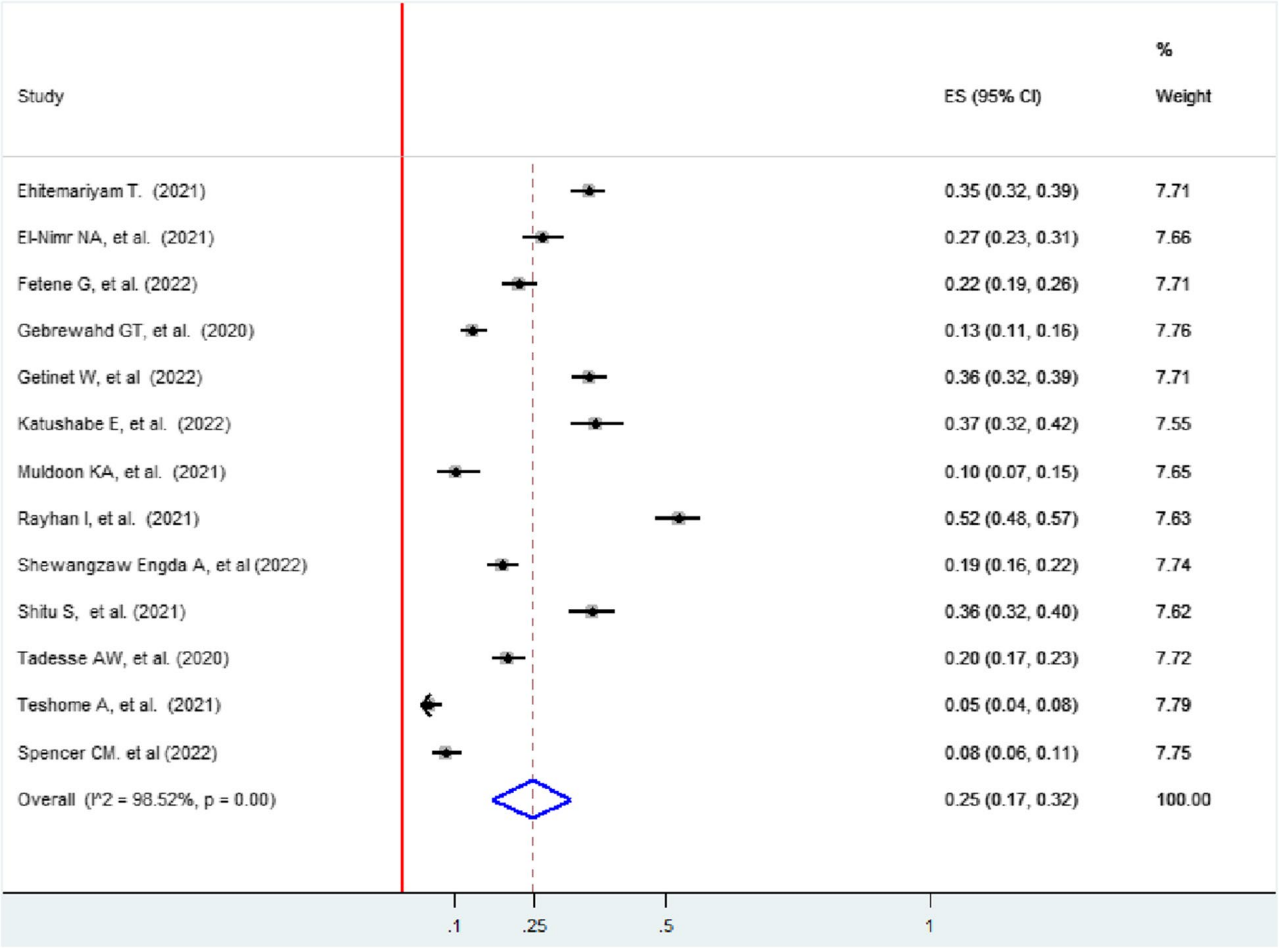
Forest plot showing the pooled prevalence of emotional violence among women during the COVID-19 pandemic

The pooled prevalence of emotional violence faced by women with intimate partners during the pandemic in 13 studies was 25% (95% CI: 17, 32) [4, 13, 40–45, 47–50, 52] (Fig. 8).

### Economic violence

**Fig. 9:**
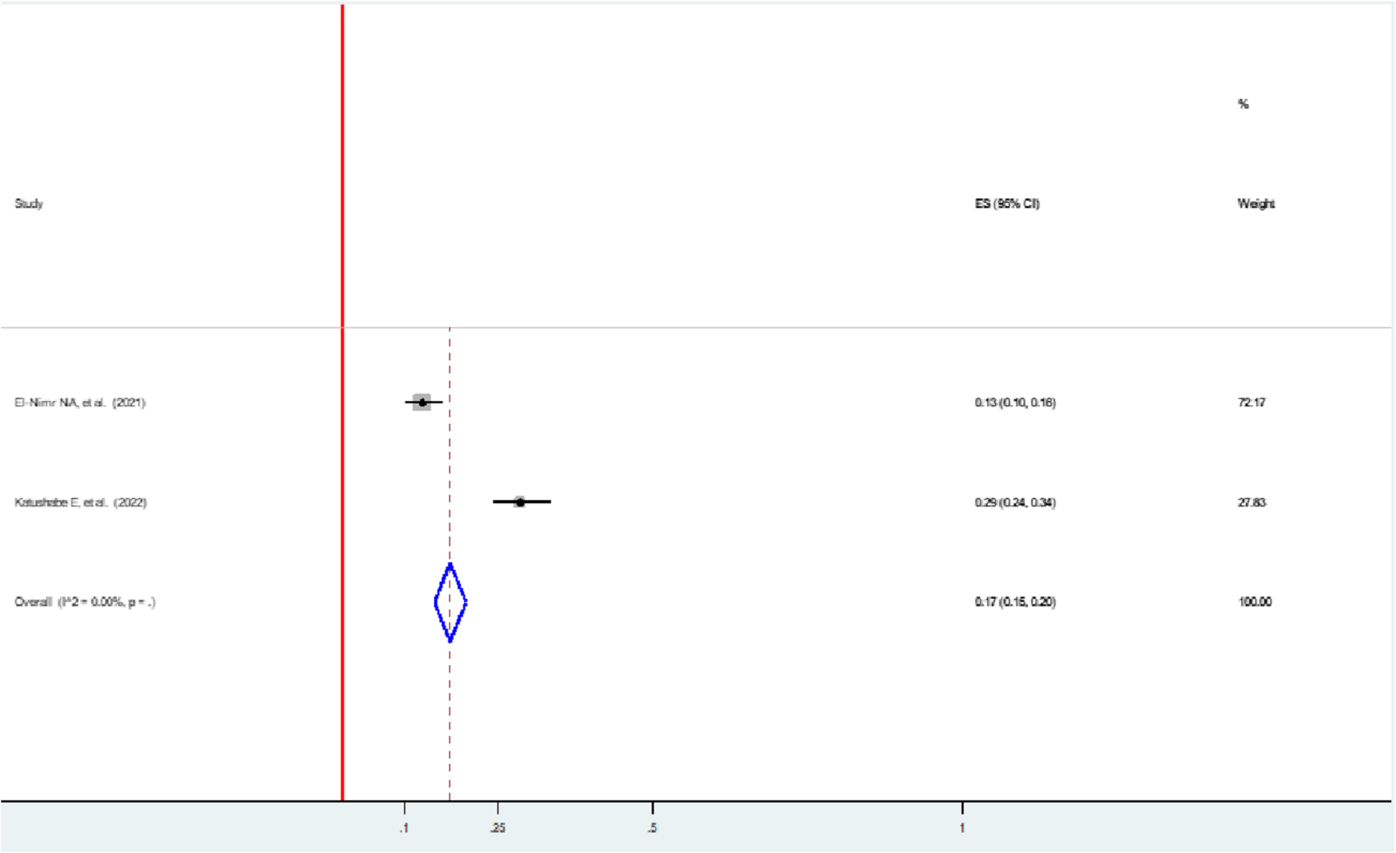
Forest plot showing the pooled prevalence of emotional violence among women during the COVID-19 pandemic

The pooled prevalence of economic violence faced by women with intimate partners during the pandemic in two studies was 17% (95% CI: 15, 20) [47, 49] (Fig. 9).

### Sexual violence

**Fig. 10:**
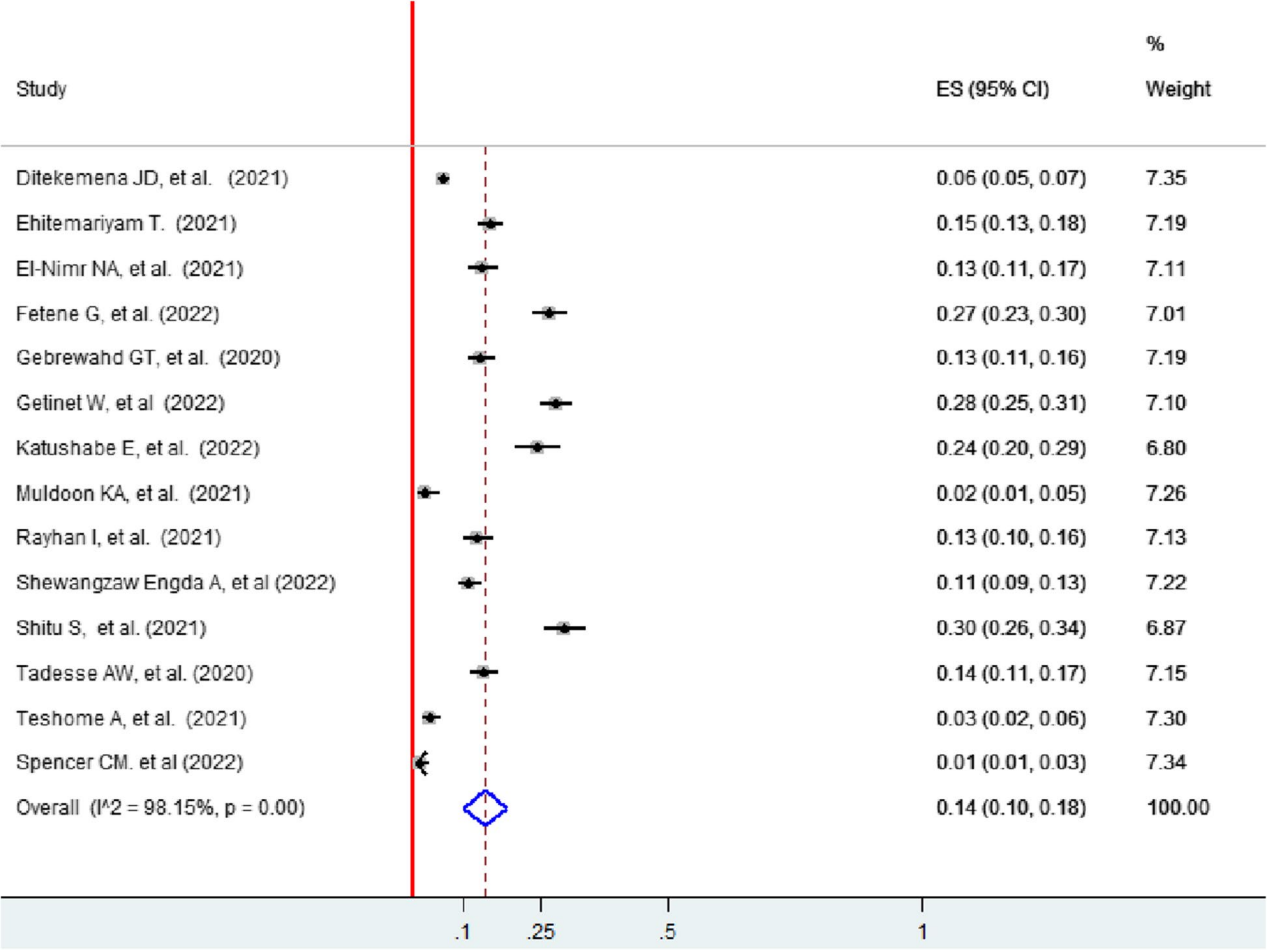
Forest plot showing the pooled prevalence of sexual violence among women during the COVID-19 pandemic

The pooled prevalence of sexual violence faced by women with intimate partners during the pandemic in 14 studies was 14% (95% CI: 10, 18) [4, 13, 40–50, 52] (Fig. 10).

### Physical violence

**Fig. 11:**
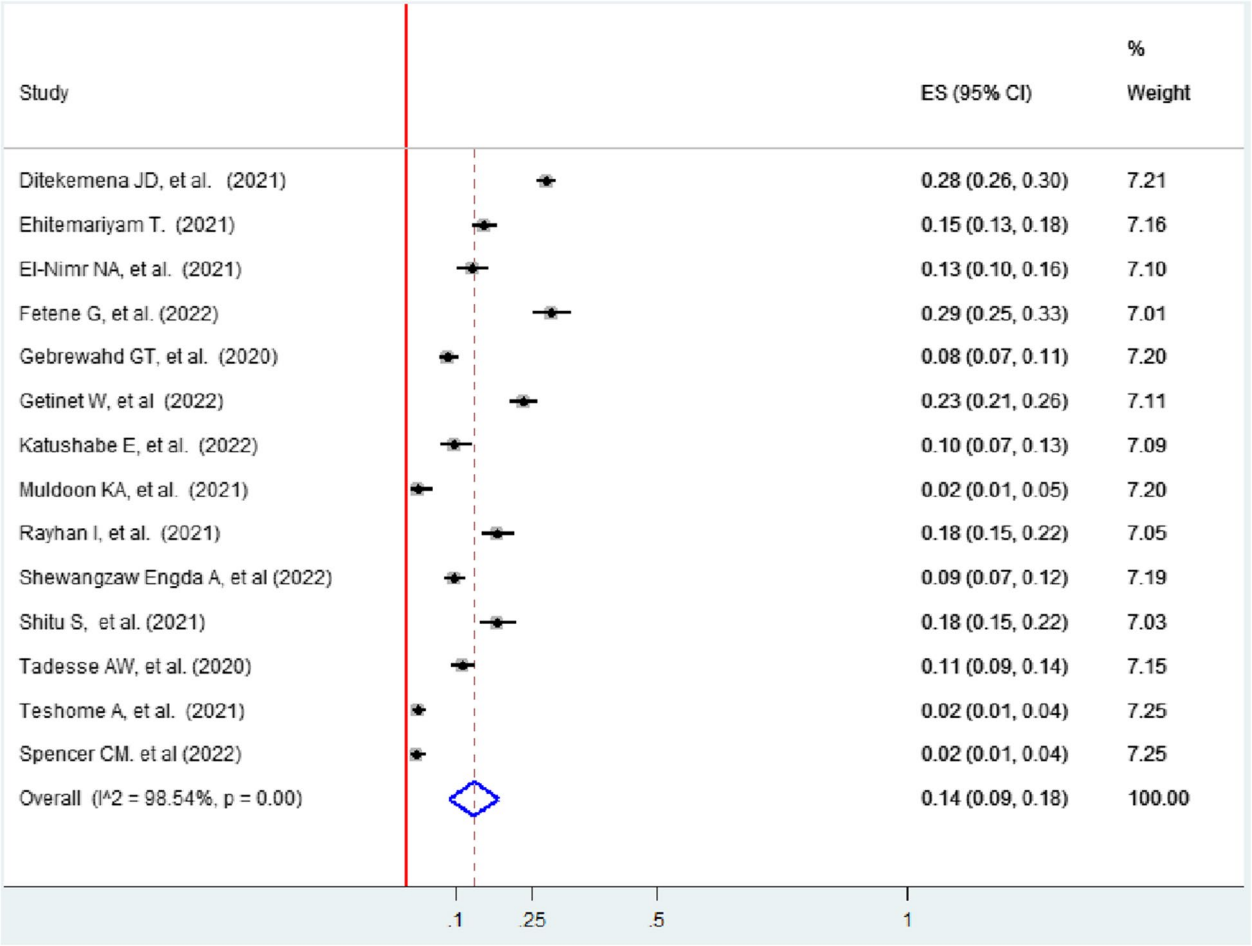
Forest plot showing the pooled prevalence of physical violence among women during the COVID-19 pandemic

The pooled prevalence of physical violence faced by women with intimate partners during the pandemic in 14 studies was 14% (95% CI: 9, 18) [4, 13, 40–50, 52] (Fig. 11).

## Discussion

This systematic review and meta-analysis aimed to estimate the pooled prevalence of intimate partner violence during the COVID-19 pandemic. To the best of our knowledge, no systematic review or meta-analysis has been conducted on the pooled prevalence of intimate partner violence during the COVID-19 pandemic. Moreover, there is a lack of representative data on intimate partner violence during the COVID-19 pandemic, and there are also inconsistent findings. Therefore, this systematic review and meta-analysis will help policy-makers, programmers, planners, clinicians, and researchers design appropriate strategies.

The pooled prevalence of any form of IPV among women during the COVID-19 pandemic was 31% (95% CI: 22–40). This prevalence was comparable to a systematic review performed before the pandemic of 30% [53] and 27% [54] and during the pandemic of 31% [55] and 33.4% [56]. However, the pooled prevalence was higher than that in studies performed before the pandemic: sub-Saharan Africa, 20% [54]; northern Africa, 15% [57]; southern Asia, 19% [14]; western Asia, 13% [7]; African countries, 15.23% [58]; China, 7.7% [59]; and France, 7% [60]. Moreover, it was also higher than studies performed in the United States, 18.0% [17], Ethiopia, 26.6% [40], and Arab countries, 22.2% [49], during the pandemic. Our finding was lower than those of a study conducted in Peru (48.0% [61]), New Orleans (59% [62]), Jordan (40% [63]), Iran (65.4% [64]), and Bangladesh (45.29% [48]). The difference might be due to differences in sample size, study setting, study period, availability, and access to health services, reproductive health information, geographical areas, and the cultures of study subjects.

In this study, the prevalence of each component of IPV during the pandemic was also determined as controlling violence, verbal violence, emotional violence, economic violence, sexual violence and physical violence, which are the prevalent forms of violence faced during the pandemic by women with intimate partners.

The limitation of this study is that it includes only articles published in the English language. Databases such as Scopus and EMBASE were not considered due to the lack of free access, and we recommend funding to expand the database search source. Additionally, all included studies in this meta-analysis were cross-sectional; as a result, the outcome variables could be affected by other confounding variables, and cause and effect relationships could not be determined. Furthermore, studies from seven countries fulfilled the eligibility criteria and may not be representative. Despite these limitations, searching, selection and data extraction of the studies were performed based on eligibility criteria independently by two authors, and ambiguity was resolved by a third author.

## Conclusions

Nearly one in three women experienced intimate partner violence during the COVID-19 pandemic. Subgroup analysis based on region showed that the highest prevalence of intimate partner violence was in developing regions (33%). All forms of intimate partner violence (physical, sexual, emotional, and economic) were prevalent. Thus, available interventions should be implemented to alleviate women’s intimate partner violence during the COVID-19 pandemic and similar emerging and remerging pandemics, particularly in developing countries.

### Supplementary Information

**Additional file 1.**

**Additional file 2.**

**Additional file 3.**

**Additional file 4.**

**Additional file 5.**

## Data Availability

All data generated or analysed during the current study are included in this manuscript and its supplementary information files.

## Abbreviations

COVID-19: Coronavirus
CINHAL: Cumulative Index to Nursing and Allied Health Literature
IPV: Intimate Partner Violence
MeSH: Medical Search Headings
PRISMA: Preferred Reporting Items for Systematic Review & Meta-analysis
RevMan: Review Manager Software
