# Supplementary material for: Global prevalence of intimate partner violence during the COVID-19 pandemic among women: systematic review and meta-analysis
Source: BMC Womens Health. 2024 Feb 17;24:127. doi: 10.1186/s12905-023-02845-8 (PMC10874578; doi:10.1186/s12905-023-02845-8)
Supplement: Supplementary file 2 — Additional file 2. [file 12905_2023_2845_MOESM2_ESM.docx]

# Supplementary file 2:Database searching algorithm

**PubMed**

((((((((((((((((((((((((((((((((((((((((((((((((Intimate Partner Violence[MeSH Terms]) OR (Intimate Partner Violence[Title/Abstract])) OR (Partner Violence, Intimate[Title/Abstract])) OR (Violence, Intimate Partner[Title/Abstract])) OR (Intimate Partner Abuse[Title/Abstract])) OR (Abuse, Intimate Partner[Title/Abstract])) OR (Partner Abuse, Intimate[Title/Abstract])) OR (Dating Violence[Title/Abstract])) OR (Violence, Dating[Title/Abstract])) OR (COVID-19[MeSH Terms])) OR (COVID-19[Title/Abstract])) OR (COVID 19[Title/Abstract])) OR (SARS-CoV-2 Infection[Title/Abstract])) OR (Infection, SARS-CoV-2[Title/Abstract])) OR (SARS CoV 2 Infection[Title/Abstract])) OR (SARS-CoV-2 Infections[Title/Abstract])) OR (2019 Novel Coronavirus Disease[Title/Abstract])) OR (2019 Novel Coronavirus Infection[Title/Abstract])) OR (2019-nCoV Disease[Title/Abstract])) OR (2019 nCoV Disease[Title/Abstract])) OR (2019-nCoV Diseases[Title/Abstract])) OR (Disease, 2019-nCoV[Title/Abstract])) OR (COVID-19 Virus Infection[Title/Abstract])) OR (COVID 19 Virus Infection[Title/Abstract])) OR (COVID-19 Virus Infections[Title/Abstract])) OR (Infection, COVID-19 Virus[Title/Abstract])) OR (Virus Infection, COVID-19[Title/Abstract])) OR (Coronavirus Disease 2019[Title/Abstract])) OR (Disease 2019, Coronavirus[Title/Abstract])) OR (Coronavirus Disease-19[Title/Abstract])) OR (Coronavirus Disease 19[Title/Abstract])) OR (Severe Acute Respiratory Syndrome Coronavirus 2 Infection[Title/Abstract])) OR (SARS Coronavirus 2 Infection[Title/Abstract])) OR (COVID-19 Virus Disease[Title/Abstract])) OR (COVID 19 Virus Disease[Title/Abstract])) OR (COVID-19 Virus Diseases[Title/Abstract])) OR (Disease, COVID-19 Virus[Title/Abstract])) OR (Virus Disease, COVID-19[Title/Abstract])) OR (2019-nCoV Infection[Title/Abstract])) OR (2019 nCoV Infection[Title/Abstract])) OR (2019-nCoV Infections[Title/Abstract])) OR (Infection, 2019-nCoV[Title/Abstract])) OR (COVID19[Title/Abstract])) OR (COVID-19 Pandemic[Title/Abstract])) OR (COVID 19 Pandemic[Title/Abstract])) OR (Pandemic, COVID-19[Title/Abstract])) OR (COVID-19 Pandemics[Title/Abstract])) AND ((((((((Women[MeSH Terms]) OR (Women[Title/Abstract])) OR (Girls[Title/Abstract])) OR (Girl[Title/Abstract])) OR (Woman[Title/Abstract])) OR (Women's Groups[Title/Abstract])) OR (Women Groups[Title/Abstract])) OR (Women's Group[Title/Abstract]))) AND (((((((((((((((((((((((((((((Epidemiologic Factors[MeSH Terms]) OR (Epidemiologic Factors[Title/Abstract])) OR (Epidemiologic Factor[Title/Abstract])) OR (Factor, Epidemiologic[Title/Abstract])) OR (Epidemiologic Determinant[Title/Abstract])) OR (Determinant, Epidemiologic[Title/Abstract])) OR (Determinants, Epidemiologic[Title/Abstract])) OR (Epidemiologic Determinants[Title/Abstract])) OR (Factors, Epidemiologic[Title/Abstract])) OR (Risk Factors[MeSH Terms])) OR (Risk Factors[Title/Abstract])) OR (Factor, Risk[Title/Abstract])) OR (Risk Factor[Title/Abstract])) OR (Social Risk Factors[Title/Abstract])) OR (Factor, Social Risk[Title/Abstract])) OR (Factors, Social Risk[Title/Abstract])) OR (Risk Factor, Social[Title/Abstract])) OR (Risk Factors, Social[Title/Abstract])) OR (Social Risk Factor[Title/Abstract])) OR (Health Correlates[Title/Abstract])) OR (Correlates, Health[Title/Abstract])) OR (Population at Risk[Title/Abstract])) OR (Populations at Risk[Title/Abstract])) OR (Risk Scores[Title/Abstract])) OR (Risk Score[Title/Abstract])) OR (Score, Risk[Title/Abstract])) OR (Risk Factor Scores[Title/Abstract])) OR (Risk Factor Score[Title/Abstract])) OR (Score, Risk Factor[Title/Abstract]))

**Google scholar**

allintitle: "Intimate partner violence" "COVID-19"

**CINAHL**

(MM "Violence+") OR (MM "Domestic Violence+") OR (MM "Intimate Partner Violence") OR (MM "Coronavirus Infections+") OR (MM "COVID-19") OR (MM "COVID-19 Pandemic") OR (MM "Coronavirus+") OR (MM "SARS-CoV-2")
