# Supplementary material for: Global prevalence of intimate partner violence during the COVID-19 pandemic among women: systematic review and meta-analysis
Source: BMC Womens Health. 2024 Feb 17;24:127. doi: 10.1186/s12905-023-02845-8 (PMC10874578; doi:10.1186/s12905-023-02845-8)
Supplement: Supplementary file 3 — Additional file 3. [file 12905_2023_2845_MOESM3_ESM.docx]

**Supplementary file 3: Joanna Briggs Institute** (**JBI) critical appraisal checklist for analytical cross sectional studies.**

Reviewer ______________________________________ Date_______________________________

Author_______________________________________ Year_________ Record Number_________

|  | Yes | No | Unclear | Not applicable |
| --- | --- | --- | --- | --- |
| 1. Were the criteria for inclusion in the sample clearly defined? | □ | □ | □ | □ |
| 1. Were the study subjects and the setting described in detail? | □ | □ | □ | □ |
| 1. Was the exposure measured in a valid and reliable way? | □ | □ | □ | □ |
| 1. Were objective, standard criteria used for measurement of the condition? | □ | □ | □ | □ |
| 1. Were confounding factors identified? | □ | □ | □ | □ |
| 1. Were strategies to deal with confounding factors stated? | □ | □ | □ | □ |
| 1. Were the outcomes measured in a valid and reliable way? | □ | □ | □ | □ |
| 1. Was appropriate statistical analysis used? | □ | □ | □ | □ |

Overall appraisal: Include □ Exclude □ Seek further info □

Comments (Including reason for exclusion)

________________________________________________________________________________________________________________________________________________________________________________________________________________________________________________________________________________________________
