# Supplementary material for: Global prevalence of intimate partner violence during the COVID-19 pandemic among women: systematic review and meta-analysis
Source: BMC Womens Health. 2024 Feb 17;24:127. doi: 10.1186/s12905-023-02845-8 (PMC10874578; doi:10.1186/s12905-023-02845-8)
Supplement: Supplementary file 4 — Additional file 4. [file 12905_2023_2845_MOESM4_ESM.docx]

**Supplementary file 4: Appraisal of included studies based on Joanna Briggs Institute** (**JBI) critical appraisal tools.**

| Studies | **Q1** | **Q2** | **Q3** | **Q4** | **Q5** | **Q6** | **Q7** | **Q8** | **RESULT** |
| --- | --- | --- | --- | --- | --- | --- | --- | --- | --- |
| Cannon et  al (2021)([76](#_ENREF_76)) | **Yes** | **Yes** | **Yes** | **Yes** | **Na** | **Na** | **Yes** | **Yes** | **Yes=6**  **Na=2** |
| Ditekemena  JD et al(2021)([71](#_ENREF_71)) | **Yes** | **Yes** | **Yes** | **Yes** | **Yes** | **Yes** | **Yes** | **Yes** | **Yes=10** |
| EHITEMARIYAM (2021)([4](#_ENREF_4)) | **Yes** | **Yes** | **Yes** | **Yes** | **Yes** | **Yes** | **Yes** | **Yes** | **Yes=10** |
| El-Nimr NA  et al(2021)([74](#_ENREF_74)) | **Yes** | **Yes** | **Yes** | **Yes** | **No** | **Yes** | **Yes** | **Yes** | **Yes=7**  **No=1** |
| G Fetene  et al (2022)([65](#_ENREF_65)) | **Yes** | **Yes** | **Yes** | **Yes** | **Na** | **Na** | **Yes** | **Yes** | **Yes=6**  **Na=2** |
| Gebrewahd  GT et al(2020)([64](#_ENREF_64)) | **Yes** | **Yes** | **Yes** | **Yes** | **Yes** | **Yes** | **Yes** | **Yes** | **Yes=10** |
| Katushabe E  et al (2022)([72](#_ENREF_72)) | **Yes** | **Yes** | **Yes** | **Yes** | **No** | **No** | **No** | **Yes** | **Yes=5**  **No=3** |
| Muldoon  KA et al (2021)([75](#_ENREF_75)) | **Yes** | **Yes** | **Yes** | **Yes** | **Yes** | **Yes** | **Yes** | **Yes** | **Yes=10** |
| Wondale Getnet et al (2022)([66](#_ENREF_66)) | **Yes** | **Yes** | **Yes** | **Yes** | **Yes** | **Yes** | **Yes** | **Yes** | **Yes=10** |
| Rayhan I &Khaleda Akter (2021)([73](#_ENREF_73)) | **Yes** | **Yes** | **Yes** | **Yes** | **Na** | **Na** | **Yes** | **Yes** | **Yes=6**  **Na=2** |
| Shewangzaw Engda A et.al(2022)([67](#_ENREF_67)) | **Yes** | **Yes** | **Yes** | **Yes** | **Yes** | **No** | **Yes** | **Yes** | **Yes=7**  **No=1** |
| Shitu S et al (2021)([68](#_ENREF_68)) | **Yes** | **Yes** | **Yes** | **Yes** | **Yes** | **Yes** | **Yes** | **Yes** | **Yes=8** |
| Tadesse AW  et al (2020)([69](#_ENREF_69)) | **Yes** | **Yes** | **Yes** | **Yes** | **Yes** | **Yes** | **Yes** | **Yes** | **Yes=10** |
| Teshome A  et al (2021**)(**[**70**](#_ENREF_70)**)** | **Yes** | **Yes** | **Yes** | **Yes** | **Yes** | **Yes** | **Yes** | **Yes** | **Yes=10** |
