# Supplementary figures and images for: Global prevalence of intimate partner violence during the COVID-19 pandemic among women: systematic review and meta-analysis
Source: BMC Womens Health. 2024 Feb 17;24:127. doi: 10.1186/s12905-023-02845-8 (PMC10874578; doi:10.1186/s12905-023-02845-8)

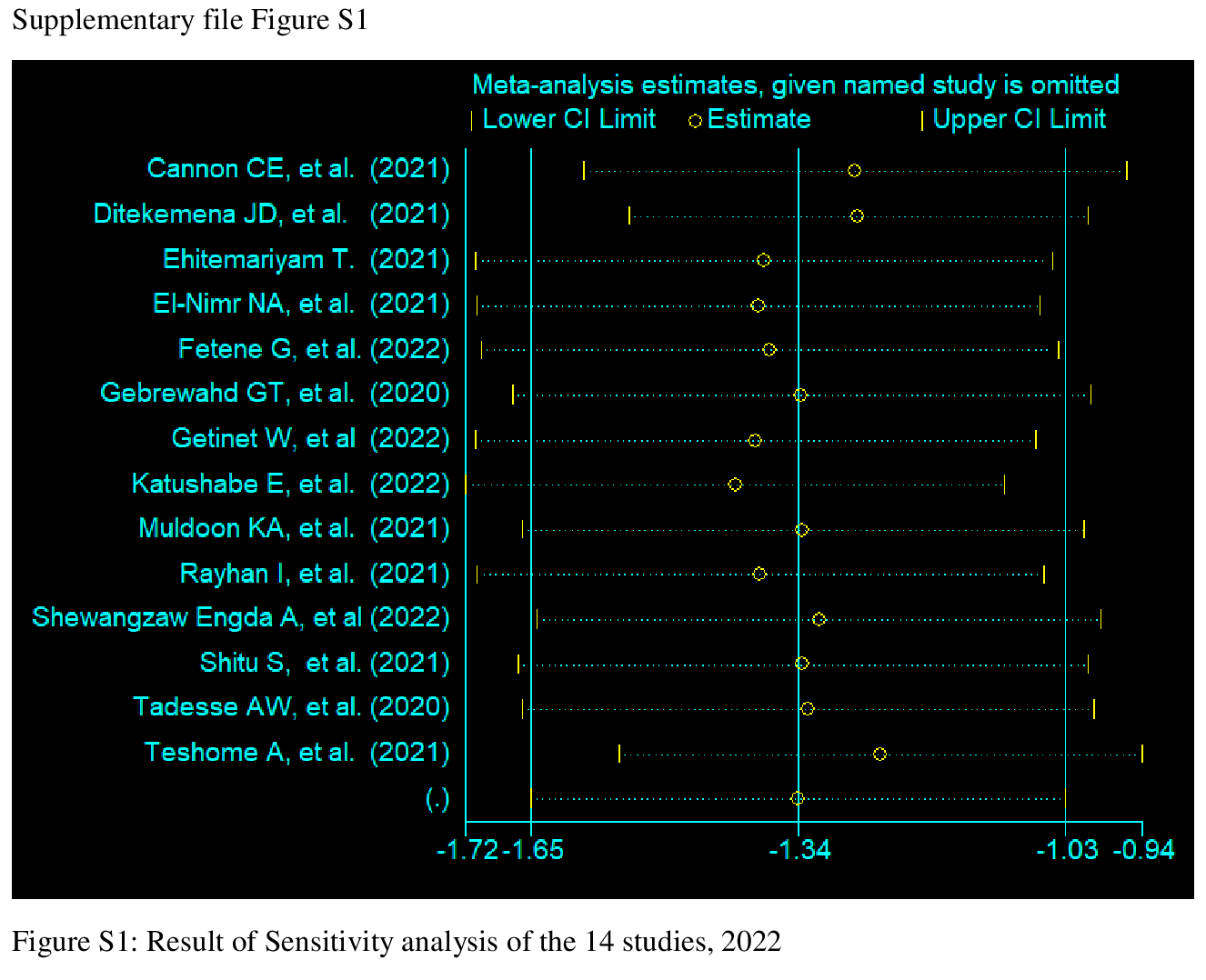


Supplementary figure file -S1

Supplement: Supplementary file 5 — Additional file 5. [file 12905_2023_2845_MOESM5_ESM.docx]
